# Prevalence of multiple lung diseases among the people and associated with wealth status and behavior factors: a longitudinal study in West Bengal

**DOI:** 10.1186/s41043-025-00917-z

**Published:** 2025-11-25

**Authors:** Ujjwal Das, Nishamani Kar, Gowthamm Mandala, Sanju Purohit

**Affiliations:** 1 P.G. Department of Geography, Fakir Mohan University, Balasore, Odisha India; 2 Department of Geography, Rajiv Gandhi University, Itanagar, Arunachal Pradesh India; 3 Purdue University, West Lafayette, IN USA; 4 Department of Environmental/Ecological Studies and Sustainability, Akamai University, Kamuela, USA

**Keywords:** Alcohol, Binary logistic regression, Inequality, Life table, Rich-poor ratio, Smoking, Wealth

## Abstract

**Background:**

Chronic lung diseases pose a significant public health challenge in India. The present study aims to examine the prevalence of lung diseases in relation to wealth status and behavioural factors among adults in West Bengal.

**Methods:**

This study utilized data from the first wave of the Longitudinal Ageing Study in India (LASI), conducted in 2017–18. The outcome variable was the presence of lung diseases. The key explanatory variables included household wealth status and behavioural factors such as alcohol, smoking, and tobacco consumption. To measure inequality, the rich-poor ratio and rich-poor difference were calculated based on Monthly Per Capita Expenditure (MPCE) quintiles. Binary logistic regression was employed to assess the association between chronic lung diseases and various socio-demographic and health-related factors. Additionally, life table techniques were used to estimate the total number of years of life lost due to alcohol, smoking, and tobacco consumption across different wealth categories.

**Results:**

The prevalence of lung diseases among individuals aged above 75 years was 17%. Prevalence was higher among older males than females (15% vs. 8%). Among those who frequently consumed alcohol, smoked, and used tobacco, the prevalence rates were 8%, 10%, and 21%, respectively. The risk of lung diseases was higher among uneducated individuals compared to those with education (OR = 1.46 vs. OR = 0.63). The total years of life lost due to lung diseases by age 75 ranged from 10.9 years (CI 11.5–13.2) in the poorest quintile (Q1) to 25.3 years (CI 24.8–25.9) in the richest quintile (Q5).

**Conclusion:**

Given the higher prevalence of lung diseases among older adults, health programs should aim to increase awareness and address substance use, particularly alcohol consumption, smoking, and tobacco use among vulnerable populations. Strengthening lung health initiatives tailored to behavioural and socio-economic risk factors is essential for improving health outcomes.

## Introduction

Most people understand how smoking causes cancer, but the aetiology of lung cancers is not very well known by everyone in society. The prevalence of lung diseases was higher in the developed nations, but in recent years, it has been increasing substantially in the developing countries [1]. Although the worldwide population is affected by chronic lung diseases, the age-sex structure of these diseases is more concentrated in age groups above 60 years [2]. Throughout the world, 235 million of the people have asthma and 251 million have chronic obstructive pulmonary diseases [3]. Incidence of these diseases 6% concentrated in age groups 50 years and below, 21% covered in age groups 50–60 years, 29% between the age 60–69 years, and 44% among the patients 70 years and older [4]. Age has strongly determined the lung disease mortality rate; with the highest numbers of mortality found in the older age groups, irrespective of their sex [5]. Lung cancer is the second leading cause of death worldwide, followed by breast cancer in women, with an estimated 2.2 million new cases and 1.8 million deaths in 2020 [6]. A developed country such as the USA reported that less than 0.5% of deaths occur due to lung cancers among the younger ages (less than 40 years) and this incidence rate rises steeply around age 45–49 year and peaks at the 85–89 years age group for males and 80–84 years for females [7]. The incidence rate was higher among the males than the females in aged 55–59 years [8]. While in Turkey cancer statistics published that lung cancer is the most common type of cancer and contributed to the highest number of deaths among the males and the fifth most common cancer among the females [9].

Wealth status plays a crucial role in shaping health outcomes, as it influences healthcare access, nutritional status, living conditions, and exposure to risk factors such as indoor pollution and smoking [10]. Individuals belonging to lower wealth strata may experience a disproportionate burden of lung diseases due to limited access to preventive and curative healthcare services, unhealthy living conditions, and greater exposure to environmental pollutants [11].

An established review suggested that smoking, tobacco and alcohol consumption contributed to 90–95% of lung diseases [12–17]. More than 50 diseases are caused by high-level smoking consumption among the people [18]. Globally, smoking among males was 47% and females 12% respectively. Available data suggested that in developing countries males formed the higher number of smokers than females, 48% vs.7%, while in developed countries, males’ percentage of smoking was 42% and females’ 24% respectively [19]. In the USA, cigarette smoking accounts for 81.7% of cases of lung cancer and 81.3% in lung cancer deaths [20]. In India, one of the middle developing countries, the highest number of smoking and tobacco consumption was found in different geographic regions as the people have used tobacco in different forms such as *beedi, cigarette, hookah, and cigar*; chewable tobacco like *gutka, khaini*; applying tobacco such as *gul* and others forms” [19]. India has contributed to the highest number of oral cancers worldwide through the widespread use of chewing tobacco [21]. According to the World Health Organization (WHO), in India, more than 8 million people have died and 12 million people are ill every year by using tobacco [22]. The cancer mortality rate increased from 683,800 in 2014 to 784,821 in 2018 [23, 24]. According to the World Health Report 2002, in India 56–80% of chronic respiratory diseases and 22% of cardiovascular diseases are as a result of high levels smoking [25]. In addition, there are some evidence that suggests that the risk of lung cancer is strongly associated with high levels of alcohol consumption. Worldwide, “1.8 million deaths and 58.3 million new cases daily are attributed to excessive amounts of alcohol consumption” [19]. On the contrary, some of the studies have found that alcohol is not an important risk factor for lung cancers [26, 27]. In many low and middle-income countries, the use of cooking fuel, biomass, and stove for cooking and heating, is an emerging risk factor for lung cancer [28]. Low socio-economic households have a higher prevalence of lung diseases than the households belonging to higher economic conditions. This socio-economic dynamic is quite common in India, where 70% of the household population belonged to rural residents as compared to 30% of urban residents.

The prevalence of lung diseases varies across socio-economic conditions and geographical regions in India. Age-adjusted lung disease incidence ranges from 44 per 100,000 male population in rural Maharashtra to 121 in Delhi [29]. Krishnamurty et al. (2012) reported a high prevalence of lung disease in Chennai, which was primarily attributed to bidi and cigarette smoking, particularly among men [30]. In Kerala, tobacco and alcohol consumption have been identified as major risk factors for gastric cancer [31]. Similarly, a study by Sehgal et al. (2012) on oesophageal cancer in Jammu suggested that long-term survival probabilities were significantly lower among individuals who consumed tobacco in the form of snuff or smoking compared to non-users [32]. Previous studies have extensively documented the burden of lung diseases among the elderly, emphasizing the significant influence of socio-economic and behavioural factors on health outcomes [33]. Lower wealth status has been linked to a higher prevalence of chronic respiratory conditions due to inadequate access to healthcare, poor living conditions, and prolonged exposure to environmental pollutants such as indoor air pollution from solid fuel use [34, 35]. Studies from low- and middle-income countries, including India, have established a strong association between household air pollution and respiratory diseases, with elderly individuals being particularly vulnerable due to lifelong exposure [36].

The percentage of elderly individuals consuming tobacco and smoking, including bidi and cigarettes, is among the highest in West Bengal [37]. However, there remains a gap in understanding the complex interplay between socio-economic disparities, behavioural risk factors, and lung disease prevalence in this population. This longitudinal study in West Bengal aims to provide empirical evidence on these interactions, offering insights into temporal trends and causal relationships. The findings will be crucial for public health planning and intervention strategies aimed at addressing the growing burden of lung diseases among the aging population. Therefore, the present study seeks to identify the prevalence of multiple lung diseases among individuals aged 45 years and above and their association with household wealth status and behavioural characteristics.

## Data & methods

The present study data has been used from the first wave of the Longitudinal Ageing Study in India (LASI), which was conducted in 2017–18 [38]. LASI is the first longitudinal aging survey data in India; it provides information on economics, health, different healthcare policies, and social drivers of population aging in India. A total of 72,000 samples of old age groups aged 45 years and above and their spouses were successfully interviewed in the survey. The survey was done through the multistage stratified cluster sampling method, where in rural areas data was collected through a three-stage sampling method and urban areas a four-stage method. In the initial stage, a Primary Sampling Unit (PSU) was selected, after which villages were selected in rural areas and wards were selected in the urban areas (LASI, 2020). In the third stage families from both rural and urban areas were randomly chosen in various communities from the Census Enumeration Block (CEB). Finally, households were chosen from each CEB in the residence (LASI, 2020). The present study covered self-reported information on lung diseases with a total of 3933 adults aged 45 years and above (rural 1971 and urban 1962). Among them, 320 people were diagnosed with lung diseases.

### Variable description

#### Dependent variable

The main dependent variable was used for the study of lung disease prevalence among people 45 years and above. The first person to be diagnosed with lung diseases confirmed by an MBBS doctor, Nurse, Ayurvedic/Unani/Homeopathic/Siddha, or any trend medical specialist. If the people have no diagnosis, that means they have not suffered from lung diseases, if the people suffer from only one disease of the lung that is considered single lung diseases, and if the people suffer more than two diseases, this is considered multiple lung diseases which include Asthma, Bronchitis, COPD (chronic obstructive pulmonary disease) and others specified.

#### Independent variable

As established, the reviewed literature suggests that several socio-demographic and lifestyle factors have a significant effect on the prevalence of lung disease among the adult population [39–42]. The operational definitions of this variable include socio-economic and demographic variables such as: sex of the respondents (male; female), age (45–54, 55–64, 65–69, and 70 + years), place of residence (rural; urban), religion (Hindu; Muslim; Christian; others), caste (SC: Scheduled Caste; ST: Scheduled Tribe; OBC: Other Backward Class; others), marital status (currently married; widowed; others), education (no education; primary; secondary; higher secondary), working status (currently working; not currently working; never worked), and Monthly Per Capita Expenditure (MPCE) quintile (poorest; poorer; middle; richer; richest).

Lifestyle factors included in the study were alcohol consumption (yes; no), tobacco consumption (yes; no), and smoking (yes; no).

### Statistical methods

Descriptive statistics such as frequency and percentage were used for the present study. To examine the association between outcome and explanatory variables chi-square test was used in the study. The binary logistic regression analyses were used to identify the odd ratios (Ors) for multiple lung diseases with adjusted outcome variable (i.e., lung diseases) and explanatory variables (i.e., age, sex, marital status, education, religion, caste, wealth status, working status, alcohol, tobacco, and smoking consumption) with 95% confidence intervals in the study. The equation of binary logistic regression is given below,

$$\log \left( {\frac{{p_{i} }}{{1 - p_{i} }}} \right) = \log it\left( {p_{i} } \right) = \beta_{0} + \beta_{1} x_{1} + \beta_{2} x_{2} + \cdots \beta_{n} x_{n}$$

In the above regression equation, ${\text{p}}_{\text{I}}$ is the probability of being perceived as single or multiple lung diseases, ${\text{x}}_{1},{\text{x}}_{2}{\dots \text{x}}_{\text{n}}$ are the predictors, ${\upbeta }_{0}$ is the intercept and ${\upbeta }_{1}$, ${\upbeta }_{2}$…${\upbeta }_{\text{n}}$ are the coefficient.

And finally, the life table technique was developed for analysing the total number of years lost due to substance use of alcohol, tobacco, and smoking along with their household wealth status. All the analysis has been done by statistical software STATA version 14.2 respectively.

## Results

Table 1 shows the sample distribution amongst people with socio-demographic and behavioural characteristics. A total of 3933 participated in the state of West Bengal, among them 1620 (41.19%) are males and 2313 females (58.81%). The majority of the study population was aged 45–54 years (45.93%). Among them, 49.9% of the population belonged to urban residences and 50% belonged to rural residences respectively. Overall, 75.82% of the participants were married and 39.55% had no formal education. Most of the study population was Hindu (79%), followed by Muslim and the percentage of Schedule Cast (SC) and Schedule Tribe (ST) were 25% and 4% respectively. In this state, the majority of the household belonged to the poorest to middle economic background family and only 15.74% belonged to the richest quintile respectively. Looking into behavioural characteristics percentage of substance alcohol, smoking, and tobacco consumption among the sample were 11.79%, 62.23%, and 37.51% respectively.

**Table 1:** Socio-demographic profile of the study area

Background characteristics	N	%
*Age*		
< 45	499	12.69
45–54	1,338	34.02
55–64	1,042	26.49
65–74	671	17.06
75 +	383	9.74
*Sex*		
Male	1620	41.19
Female	2313	58.81
*Marital status*		
Currently married	2982	75.82
Widowed	811	20.62
Others	140	3.56
*Education*		
No education	1555	39.55
< 5	561	14.27
5–9 Years	1001	25.46
> 10 years more	815	20.73
*Visiting health care facility*		
Yes	416	10.64
No	3495	89.36
*Religion*		
Hindu	3122	79.38
Muslim	754	19.17
Christian	19	0.48
*Social groups*		
Schedule cast	971	24.77
Schedule tribe	172	4.39
Other backward class	433	11.05
Others	2344	59.8
*Working status*		
Currently working	1754	44.64
Not working cur	833	21.2
Never worked	1342	34.16
*MPCE quintile*		
Poorest	691	17.57
Poorer	943	23.98
Middle	869	22.1
Richer	811	20.62
Richest	619	15.74
*Alcohol consumption*		
Yes	461	11.79
No	3448	88.21
*Tobacco consumption*		
Yes	1467	37.51
No	2444	62.49
*Smoking consumption*		
Yes	2434	62.23
No	1477	37.76

**Fig. 1:**
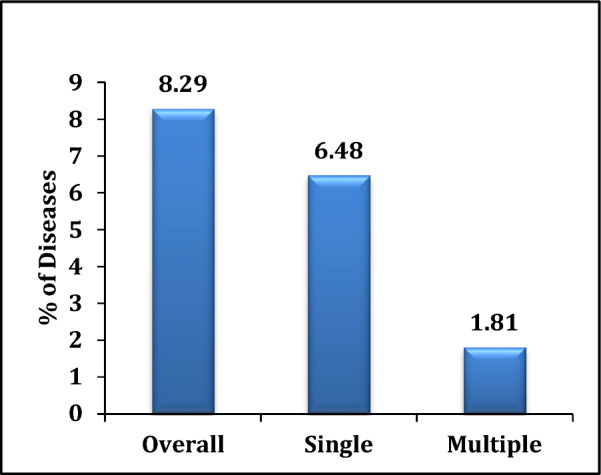
Prevalence of lung diseases

**Fig. 2:**
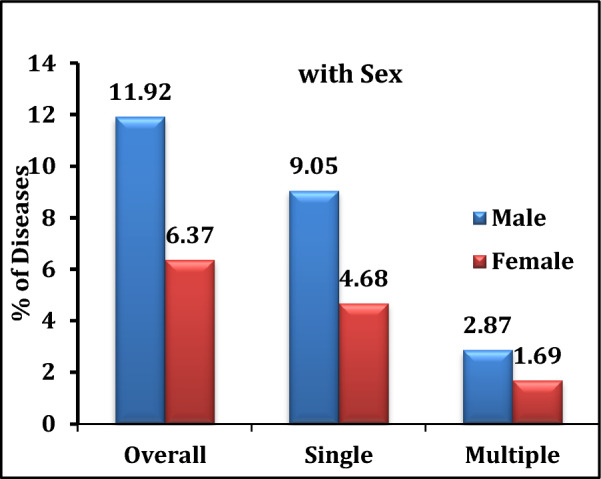
Prevalence of lung diseases with sex

Table 2 presents the prevalence of lung diseases among older adults across various background characteristics. It was observed that the prevalence of lung diseases increased with age. For instance, among individuals below 45 years, the prevalence was 2%, whereas it rose to 5%, 8%, 13%, and 17% in the age groups 45–54, 55–64, 65–74, and 75 years and above, respectively. Similarly, the prevalence of multiple lung diseases increased from 1.4% in the 45–54 age group to 2.9% among those aged 75 years and above. The prevalence of both single and multiple lung diseases was higher among males compared to females (11.9% vs. 6.4% and 2.9% vs. 1.7%, respectively; Fig. 2). Among social and cultural groups, higher percentages of lung disease were found among widowed individuals, those with no education or below primary education, Muslims, and members of the OBC category. Elderly individuals who were not currently working showed a higher prevalence of lung diseases compared to those who were currently working. For instance, a total of 12.9% of non-working individuals reported lung diseases—10.2% had single diseases, and 2.7% had multiple lung diseases. Older adults from both the poorest and richest economic backgrounds exhibited higher percentages of lung diseases. However, the prevalence of multiple lung diseases was lower among those from the richest background compared to the poorest (0.81% vs. 2.02%). Additionally, individuals who frequently consumed alcohol or smoked tobacco had higher prevalence rates of lung diseases—10%, 13%, and 21%—than those who did not consume alcohol, smoke, or use tobacco, respectively. Overall, the results suggest that the total prevalence of lung diseases among older adults was 8.29%, with 6.48% having single diseases and 1.81% having multiple lung diseases (Fig. 1).

**Table 2:** Prevalence of lung diseases (%) among the adults by different background characteristics, in West Bengal

Background characteristics	Overall (95% CI)	No disease	Single (95%CI)	Multiple (95%CI)	χ^2^	*P*
*Age*						
< 45	2.21 (1.57–3.01)	97.79	2.21 (1.57–3.01)	0		
45–54	5.72 (4.05–6.15)	94.28	4.35 (3.01–6.10)	1.37 (0.54–2.51)		
55–64	8.41 (7.15–9.25)	91.59	7.03 (5.21–9.45)	1.38 (0.85–2.10)	122.05	*0.000*
65–74	12.3 (8.04–15.26)	87.7	9.4 (7.10–10.10)	2.9 (1.52–3.58)		
75 +	16.8 (12.6–18.69)	83.2	12.86 (10.5–15.45)	3.94 (2.52–4.12)		
*Sex*						
Male	11.92 (10.25–15.63)	88.08	9.05 (7.84–10.21)	2.87 (1.52–3.25)	30.55	*0.000*
Female	6.37 (4.25–8.57)	93.63	4.68 (3.52–5.21)	1.69 (1.41–2.71)		
*Place of residence*						
Rural	9.69 (6.89 – 11.25)	90.31	7.62 (6.52–8.54)	2.07 (1.14–3.10)		
Urban	6.78 (4.58 – 9.56)	93.22	5.32 (4.01–6.52)	1.46 (1.02–2.11)	13.61	*0.001*
*Marital status*						
Currently married	7.77 (6.58–9.12)	92.23	6.16 (5.02–7.01)	1.61 (0.98–2.10)		
Widowed	10.12 (8.56–15.96)	89.88	7.64 (6.25–7.98)	2.48 (1.54–2.98)	10.03	*0.04*
Others	6.43 (4.69–8.45)	93.57	6.43 (4.69–8.45)	0		
*Education*						
No education	8.93 (7.45–11.36)	91.07	6.83 (5.02–7.26)	2.1 (1.52–3.01)		
Primary	11.53 (9.56–17.56)	88.46	9.46 (8.10–9.98)	2.07 (1.59–2.96)	22.25	*0.001*
Secondary	7.5 (5.8–9.87)	92.5	6 (5.10–6.89)	1.5 (0.8–2.0)		
Higher secondary	4.57 (2.56–6.58)	95.43	4.32 (3.58–5.01)	0.25 (0.11–0.98)		
*Religion*						
Hindu	7.91 (4.59–9.85)	92.09	6.14 (5.85–7.01)	1.77 (1.12–2.51)		
Muslim	9.76 (7.04–11.45)	90.24	8.1 (7.48–9.15)	1.66 (1.01–2.01)	6.75	*0.344*
Christian	5.26 (4.12–6.59)	94.74	5.26 (4.12–6.59)	0		
Others	5.26 (4.58–7.05)	94.74	2.63 (1.12–3.01)	2.63 (1.12–3.01)		
*Caste*						
SC	8.05 (6.96–10.25)	91.95	7.02 (6.12–7.85)	1.03 (0.52–1.89)		
ST	8.72 (6.12–11.23)	91.28	6.98 (5.89–7.36)	1.74 (1.02–2.10)	7.77	*0.255*
OBC	8.57 (7.01–11.25)	91.44	7.41 (6.52–8.10)	1.16 (0.85–1.52)		
Others	6.59 (4.26–8.47)	93.41	6.08 (5.62–6.89)	0.51 (0.01–0.75)		
*Working status*						
Currently working	6.17 (5.01–9.84)	93.83	5.77 (4.85–6.12)	0.4 (0.21–0.98)	38.60	*0.000*
Not working currently	12.94 (10.26–15.26)	87.06	10.25 (9.85–11.25)	2.69 (1.85–3.21)		
Never worked	5.74 (4.89–6.19)	94.25	5.07 (4.85–5.89)	0.67 (0.25—0.98)		
*MPCE quintile*						
Poorest	8.42 (6.58–10.25)	90.59	6.4 (5.88–6.89)	2.02 (1.02–2.96)		
Poorer	8.22 (6.12–9.85)	91.78	6.26 (5.25–7.01)	1.96 (1.05–2.14)	2.79	*0.946*
Middle	8.37 (7.05–10.25)	91.63	6.91 (5.45–7.14)	1.46 (0.85–1.59)		
Richer	6.81 (5.02–9.85)	93.19	6.19 (5.85–7.01)	0.62 (0.21–1.12)		
Richest	7.47 (5.89–9.02)	92.53	6.66 (5.64–7.02)	0.81 (0.15–1.26)		
*Alcohol consumption*						
Yes	10.46 (8.85–11.56)	89.54	7.81 (6.50–8.25)	2.65 (1.52–3.10)	1.60	*0.447*
No	7.08 (6.15–9.85)	92.92	6.3 (5.01–7.25)	0.78 (0.52–1.12)		
*Tobacco consumption*						
Yes	13.3 (10.56–15.54)	86.7	9.41 (8.85–9.89)	3.89 (2.58–4.10)	34.11	*0.000*
No	5.41 (4.85–7.86)	94.59	4.71 (4.05–5.96)	0.7 (0.12–1.02)		
*Smoking consumption*						
Yes	20.78 (14.52–25.26)	79.22	17.48(15.02–18.85)	3.3 (2.25–3.89)	13.15	*0.001*
No	12.14 (9.96–16.24)	87.86	9.35 (8.85–10.25)	2.79 (2.14–3.15)		
All prevalence	8.29 (6.52–9.89)	91.71	6.48 (5.25–7.15)	1.81 (0.85–2.12)

Table 3 presents the gender-wise differences in the prevalence of lung diseases among adults associated with different background characteristics. The results found that gender wise differences in lung disease prevalence were varied across the various background characteristics. The prevalence of lung diseases was higher among the males compared to females. The males aged 75 years had a higher prevalence of lung diseases than the females in the same age groups. Furthermore, males with no education and socially marginalized people like Muslim and OBC had a higher percentage of lung diseases than the females in the same categories. The prevalence of lung diseases was higher among the male than the female due to alcohol, smoking, and tobacco consumption (8.81%, 12.14%, and 12.05% vs. 4.88%, 6.49%, 12.5%, and 10.5%) (Fig. 2).

**Table 3:** Gender-wise difference in lung diseases prevalence among adults by different background characteristics in West Bengal

Background characteristics	Lung diseases (N)		Lung diseases (%)		Difference
	Male	Female	Male	Female	
*Age*					
45–54	39	24	6.26	3.38	2.88
55–64	46	31	9.37	5.66	3.71
65–74	43	27	12.72	8.11	4.60
75 +	34	31	20.86	14.16	6.7
*Place of residence*					
Rural	95	76	11.66	6.59	5.07
Urban	67	48	8.38	4.15	4.23
*Marital status*					
Currently married	143	60	10.01	3.89	6.12
Widowed	14	60	11.86	8.66	3.2
Others	5	4	7.35	5.56	1.79
*Education*					
No education	52	72	11.95	6.44	5.51
Primary	38	21	14.73	6.95	7.78
Secondary	42	23	8.97	4.32	4.65
Higher secondary	30	8	6.61	2.24	4.37
*Religion*					
Hindu	120	97	9.31	5.32	3.99
Muslim	39	27	12.79	6.03	6.76
Christian	1	0	16.67	0	16.67
Others	2	0	13.33	0	13.33
*Caste*					
SC	39	40	9.95	6.92	3.03
ST	8	7	12.9	6.36	6.54
OBC	24	13	12.97	5.26	7.71
Others	91	64	9.33	4.7	4.63
*Working status*					
Currently working	86	23	7.75	3.58	4.17
Not working currently	70	30	15.95	7.67	8.28
Never worked	6	71	9.09	5.57	3.52
*MPCE quintile*					
Poorest	28	24	10.73	5.61	5.12
Poorer	39	29	10.29	5.15	5.14
Middle	36	28	10.03	5.5	4.53
Richer	27	28	7.89	6.01	1.88
Richest	32	15	11.68	4.37	7.31
*Alcohol consumption*					
Yes	37	2	8.81	4.88	3.93
No	124	122	10.46	5.4	5.06
*Tobacco consumption*					
Yes	122	30	12.14	6.49	5.65
No	39	94	6.47	5.11	1.36
*Smoking consumption*					
Yes	61	3	12.5	10.5	2.45
No	31	2	21.99	14.29	7.7

**Fig. 3:**
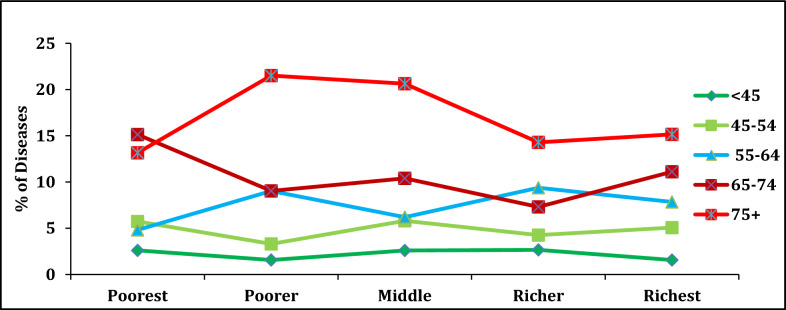
Prevalence of lung diseases with wealth status & age groups

Table 4 shows the prevalence of lung diseases among adult with their economic wealth status. Overall results show that the differences in lung disease prevalence between the poorest and richest economic background family was narrowed across the various background characteristics. The result found that the prevalence of lung diseases in people 75 years of age was higher than the 45-years age group and more so in poor background families than their rich background counterparts (Fig. 3). Another interesting result found that those people with a rich family background, had a higher percentage of lung diseases than those who belonged to the poor families due to their different lifestyles. The differencess are more marked in their behavioural characteristics (alcohol, smoking, and tobacco consumption). This might be explained by the fact that people who belonged to rich families have more purchasing power to acquire different types of alcohol, tobacco, and cigarettes. Thus, the prevalence of lung diseases was higher among the wealthier group people than the poorer group. Similarly, major differences also found in socially vulnerable groups in schedule tribe people and people with below primary education. The majority of the rich-poor ratio is more than 1 which implies that people from the richest background family are getting closer to the prevalence of lung diseases than the people from poorest background family.

**Table 4:** The percentage of lung diseases with wealth quintile among adults by different background characteristics, in West Bengal

Background characteristics	MPCE quintile (%)					Rich-poor differences	Rich-poor ratio
	Poorest	Poorer	Middle	Richer	Richest		
*Age*							
< 45	2.6	1.56	2.59	2.65	1.56	1.04	0.60
45–54	5.74	3.3	5.8	4.26	5.07	0.67	0.88
55–64	4.81	9.02	6.2	9.38	7.84	3.03	1.63
65–74	15.13	9.03	10.39	7.3	11.11	4.02	0.73
75 +	13.16	21.51	20.63	14.29	15.15	1.99	1.15
*Place of residence*							
Rural	6.60	8.93	9.60	8.84	9.38	2.78	1.42
Urban	8.71	5.59	5.00	5.16	5.72	2.99	0.66
*Sex*							
Male	10.73	10.29	10.03	7.89	11.68	0.95	1.09
Female	5.61	5.15	5.5	6.01	4.37	1.24	0.78
*Marital status*							
Currently married	8.07	6.99	6.16	6.15	7.16	0.91	0.89
Widowed	6.45	8.37	12.75	9.26	9.91	3.46	1.54
Others	5	5.26	8.11	7.14	5.88	0.88	1.18
*Education*							
No education	7.82	7.69	8.88	6.85	8.97	1.15	1.15
Primary	5.1	8.9	13.01	13.83	11.84	6.74	2.32
Secondary	8.84	7.94	3.98	6.15	6.11	2.73	0.69
Higher Secondary	5.71	0.98	3.8	4.78	6.42	0.71	1.12
*Religion*							
Hindu	7.55	7.07	6.72	6.45	7.27	0.28	0.96
Muslim	7.51	8.15	9.77	9.02	10.11	2.6	1.35
*Caste*							
SC	9.42	8.11	9.77	6.35	6.03	3.39	0.64
ST	3.08	7.32	18.75	6.67	15.79	12.71	5.13
OBC	9.62	8.82	7.92	3.9	13.64	4.02	1.42
Others	7.2	6.35	5.61	7.41	6.75	0.45	0.94
*Working status*							
Currently working	5.98	6.39	5.88	4.95	8.56	2.58	1.43
Not working currently	8.48	11.76	14.75	13.86	10.85	2.37	1.28
Never Worked	9.01	5.68	4.76	5.05	4.78	4.23	0.53
*Alcohol consumption*							
Yes	3.41	9.43	8.51	8.14	12.64	9.23	3.71
No	8.21	6.97	7.26	6.68	6.63	1.58	0.81
*Tobacco consumption*							
Yes	9.15	11.27	9.28	8.6	14.42	5.27	1.58
No	6.48	4.8	6.19	5.89	3.75	2.73	0.58
*Smoking consumption*							
Yes	9.48	14	8.05	4.46	14.42	4.94	1.52
No	13.79	12.5	30	16.57	32.35	18.56	2.35

**Fig. 4:**
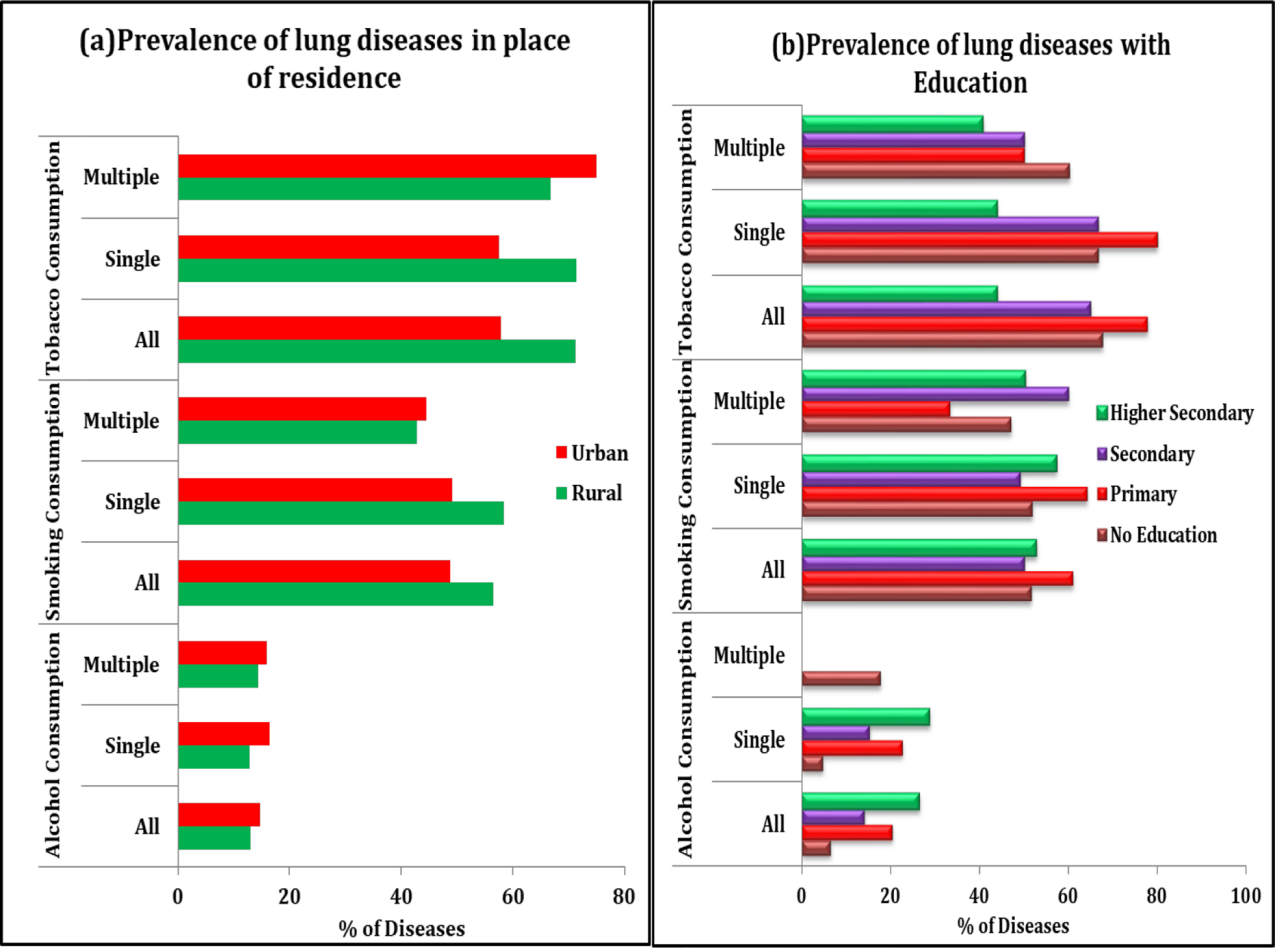
Prevalence of lung diseases with lifestyle behaviour: **a** Residence **b** Education

Table 5 describes the prevalence of lung diseases among people due to behavioural level characteristics. The results found that those people who frequently consumed alcohol, smoke cigarette, bidi, and other tobacco products, have higher prevalence of single and multiple lung diseases. The prevalence of multiple lung diseases was higher among the above 75 years aged people due to smoking and tobacco consumption than alcohol consumption (46.67%, 58.67%, and 6.67%). The overall prevalence of lung diseases was higher among the males than the female (22.98%, 75.78%, 66.30% vs. 1.61%, 24.19%, and 60.0%). Furthermore, the prevalence of multiple lung diseases was higher in urban residents due to high levels of smoking and alcohol consumption than the rural residents (Fig. 4a). Considering the education level, people with below primary education and higher secondary education had a higher prevalence of multiple lung diseases due to tobacco consumption than alcohol consumption (Fig. 4b), but the prevalence of lung diseases was lower by tobacco consumption among the educated people than the uneducated people in the state (43.75% vs. 67.65%). The Muslim people and schedule cast background people had a higher prevalence of lung diseases. The economically poorest and richest group were a higher prevalence of lung diseases due to smoking and tobacco consumption than alcohol consumption, but the prevalence of multiple lung diseases was lower among economically richest people than poorest people respectively (40.0% vs. 57.14% and 28.93% vs. 33.33%).

**Table 5:** The prevalence of lung diseases (%) with behavioural level characteristics among the older adult in West Bengal

Background characteristics	Alcohol consumption			Smoking consumption			Tobacco consumption		
	All	Single	Multiple	All	Single	Multiple	All	Single	Multiple
*Age*									
< 45	5.36	6.78	NA	9.09	9.09	NA	12.15	18.26	NA
45–54	17.46	18.97	15.32	50.79	55.17	0.00	46.67	46.67	38.63
55–64	15.58	15.07	25.00	54.94	55.75	50.00	54.19	43.33	42.37
65–74	17.39	17.74	16.67	55.07	54.84	56.67	55.52	55.38	66.67
75 +	16.15	16.12	6.67	57.69	56.94	46.67	56.63	56.33	58.67
*Sex*									
Male	22.98	24.14	14.29	75.78	77.24	64.29	66.30	66.67	71.43
Female	1.61	0.93	6.25	24.19	24.07	25.00	60.00	60.00	52.00
*Place of residence*									
Rural	12.94	12.75	14.29	56.47	58.39	42.86	71.19	71.43	66.67
Urban	14.78	16.35	15.94	48.70	49.04	44.44	57.89	57.58	75.00
*Marital status*									
Currently married	18.23	19.23	11.11	60.40	61.54	50.00	66.67	67.09	71.43
Widowed	1.35	2.35	8.33	33.78	33.87	33.33	55.56	55.56	NA
Others	11.11	11.11	NA	55.56	55.56	NA	NA	NA	NA
*Education*									
No Education	6.45	4.72	17.65	51.61	51.89	47.06	67.65	66.67	60.23
Primary	20.34	22.64	NA	61.02	64.15	33.33	77.78	80.00	50.00
Secondary	14.06	15.25	NA	50.00	49.15	60.00	65.00	66.67	50.00
Higher Secondary	26.32	28.57	NA	52.63	57.14	50.12	43.75	43.75	40.63
*Religion*									
Hindu	16.67	17.89	8.33	50.93	51.58	45.83	65.79	66.67	66.67
Muslim	3.03	3.28	2.23	62.12	63.93	40.00	66.67	65.00	60.63
*Caste*									
SC	15.19	17.65	NA	50.63	54.41	20.00	79.31	81.48	NA
ST	33.33	16.67	NA	40.00	33.33	66.67	60.23	58.39	NA
OBC	11.11	12.90	NA	66.67	74.19	20.00	66.67	66.67	NA
Others	11.61	12.68	NA	52.90	52.11	66.67	56.60	56.25	60.0
*Working status*									
Currently working	19.27	19.80	14.29	67.89	70.30	28.57	70.21	73.33	NA
Not working currently	18.18	19.05	14.29	60.61	61.90	57.14	60.47	57.89	80.00
Never Worked	14.36	18.26	12.69	23.38	22.06	33.33	71.43	66.67	NA
*MPCE quintile*									
Poorest	5.77	4.55	14.29	50.00	47.73	57.14	73.33	90.91	33.33
Poorer	14.71	15.25	11.11	58.82	61.02	44.44	84.00	82.61	30.23
Middle	12.50	13.33	14.36	48.44	50.00	25.00	57.14	57.14	25.63
Richer	12.73	14.00	18.69	43.64	44.00	40.00	50.00	50.00	22.36
Richest	23.91	25.00	20.00	67.39	72.50	40.00	57.69	54.17	28.93

NA, Not available

Table 6 shows the odds ratio of multiple lung diseases among adults with associated factors. The Model 1 presents adjusted odds ratios for lung diseases with various background characteristics. Models 2 and 3 describe the effect of wealth status and behavioural level factors in lung diseases with various socio-economic and demographic characteristics. The results of model 1 shows that increase in age substantially increased the risk of lung diseases. Female have a lower risk of lung diseases as compared to male. The people from urban residences were lower risk for lung diseases than the people who belonged to rural residence with statically significant (OR = 0.85 (0.64–1.13). People in below primary education 1.46 times higher risk of lung diseases than the higher secondary educated people (OR = 0.63 (0.39–1.01). The Muslim religion and schedule tribes’ peoples were 1.31 and 1.19 times higher for risk of lung diseases as compared to other social groups with statistically significant (OR = 1.31 (0.93–1.84) and OR = 1.19 (0.63–2.24). The people who belonged to the richest household quintile had the higher chances of lung disease as compared to other household wealth categories. The people who had frequently consumed alcohol and tobacco were at higher risks for lung diseases than those did not consume any type of alcohol or tobacco (OR = 0.68(0.85–1.92), OR = 0.70(0.52–0.93) and OR = 0.56(0.23–0.89). In Model 2 effect of wealth status on lung disease prevalence it was also found that people age 75 years and above, have higher chances of lung diseases as compared to adjusted model 1. Similarly, in below primary education, socially marginalized Muslim and scheduled tribes groups, tobacco consumption have a significant effect on lung disease Model 3 also shows the increase risks of lung diseases was due to alcohol, smoking, and tobacco consumption with their socio-economic background characteristics.

**Table 6:** Odds Ratio of multiple lung diseases among older adult by background characteristics in West Bengal

Background characteristics	Model -1		Model-2		Model-3	
	OR	95% CI	OR	95% CI	OR	95% CI
*Age*						
< 45®						
45–54	1.71**	0.87–3.37	1.72*	0.87–3.40	2.31*	0.28–19.19
55–64	2.52***	1.27–4.99	2.54***	1.28–5.02	3.94***	0.47–32.66
65–74	3.38***	1.66–6.88	3.42***	1.69–6.96	4.16***	0.50–34.89
75 +	6.06***	2.88–12.72	6.14***	2.92–12.88	8.66***	0.98–76.78
*Sex*						
Male®						
Female	0.59***	0.40–0.86	0.59***	0.40–0.89	0.39***	0.21–0.71
*Place of residence*						
Rural®						
Urban	0.85**	0.64–1.13	0.86*	0.63–1.11	0.80*	0.47–1.38
*Marital status*						
Currently married ®						
Widowed	0.96	0.67–1.37	0.98	0.67–1.40	0.80	0.43–1.48
Others	1.18	0.58–2.40	1.19	0.57–2.37	2.42	0.67–8.83
*Education*						
No Education®						
Primary	1.46**	1.03–2.07	1.52**	1.04–2.10	1.49*	0.82–2.70
Secondary	0.96	0.67–1.37	0.98	0.69–1.40	0.81	0.41–1.57
Higher Secondary	0.63*	0.39–1.01	0.69**	0.43–1.05	0.79**	0.32–1.96
*Religion*						
Hindu®						
Muslim	1.31*	0.93–1.84	1.40*	0.93–1.98	1.49*	0.80–2.77
Christian	1.06	0.13–8.28	1.04	0.11–8.19	1.04	0.132–8.21
Others	0.72	0.16–3.26	0.75	0.17–3.22	5.18	1.06–25.30
*Caste*						
SC®						
ST	1.19	0.63–2.24	1.17	0.62–2.20	1.25	0.54–2.87
OBC	0.96	0.61–1.50	0.96	0.61–1.51	1.77	0.84–3.75
Others	0.81**	0.58–1.12	0.81	0.56–1.15	1.45	0.73–2.90
*Working status*						
Currently working ®						
Not working currently	1.49**	1.07–2.08	1.52**	1.06–2.10	1.70**	0.95–3.04
Never worked	1.26*	0.85–1.86	1.21	0.85–1.96	1.86	0.92–3.77
*MPCE quintile*						
Poorest®						
Poorer	1.05	0.71–1.55			1.27	0.63–2.59
Middle	1.11	0.74–1.64			1.12	0.52–2.39
Richer	1.12	0.74–1.70			1.34	0.58–3.06
Richest	1.23	0.79–1.92			1.22	0.56–2.67
*Alcohol consumption*						
Yes®						
No	0.68*	0.85–1.92	0.61	0.85–1.91		
*Tobacco consumption*						
Yes®						
No	0.70***	0.52–0.93	0.70**	0.52–0.93		
*Smoking consumption*						
Yes®						
No	0.56***	0.23–0.89	0.59**	0.36–0.83		

**Fig. 5:**
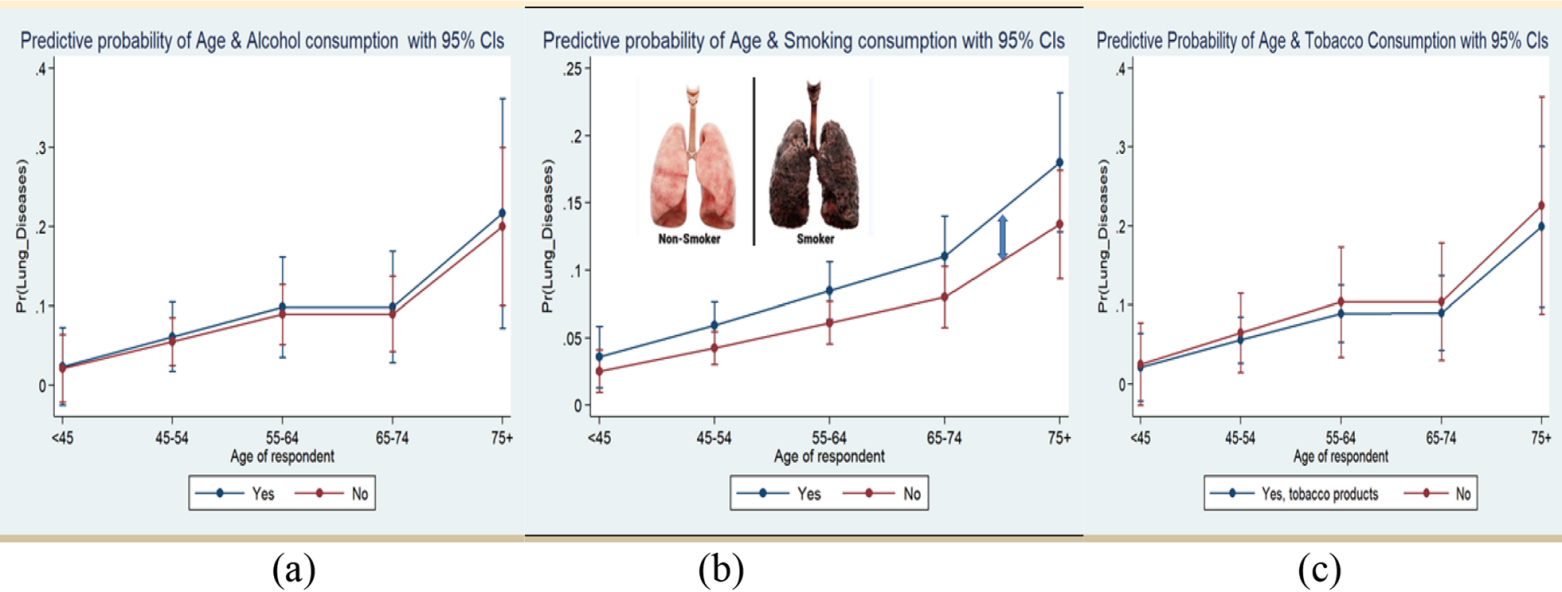
Predictive probability for risk of lung diseases **a** Alcohol **b** smoking **c** tobacco consumption

The Hosmer and Lemeshow test was used to identify the adequacy test logistic regression model (Table 7). To fit this model if the p-value is less than 0.05 then it is said to be poor. From the above table it was found that chi-square value 3.66 with 8 degrees of freedom and a p-value of 0.8864,that implies there was no significant difference between observed and predicted value and indicated that the model fit at an acceptable level. The predictive probability indicates that the prevalence of multiple lung diseases was higher among the elderly aged people due to alcohol and smoking consumption, these differences were greater in smoking consumption than the alcohol consumption (Fig. 5b).

**Table 7:** Contingency table for Hosmer and Lemeshow test

Multiple lung diseases = Yes		Multiple lung diseases = No		Total	λ	d.f	*P*
Observed	Expected	Observed	Expected				
5	6.7	385	383.3	390			
9	10.6	382	380.4	391			
15	13.6	374	375.4	389			
19	16.6	370	372.4	389			
15	20.5	375	369.5	390	3.66	8	*0.8864*
27	24.5	360	362.5	387			
27	30.0	364	361.0	391			
39	36.8	351	353.2	390			
51	48.4	338	340.6	389			
78	77.4	309	309.6	387

Table 8 shows the total number of years lost in lung diseases among the various economic background family (wealth groups) due to the smoking of tobacco and consumption. The total number of years lost at age 75 year by the MPCE wealth quintile ranged from 10.9 years (CI 11.5–13.2) for the poorest quintile (Q1) to 25.3 years (CI 24.8–25.9) for the richest quintile group (Q5). Overall gap in one disease between Q1 and Q5 was 2.6 years [Q1: 1.3 years (CI 0.9–2.3) vs. Q5: 3.9 years CI (2.6–4.2)]. While for two diseases and more than 3 diseases, there were 5.4 years and 6.4 years gap between Q1 and Q5 [Q1: 2.4 years (CI 2.0–3.1) vs. Q5: 7.8 years (CI 6.5–8.6) and Q1: 7.2 years (CI 7.5–11.2) vs. Q5: 13.6 years (CI 11.3–14.6)] The total numbers of years lost in multiple-lung diseases at age 75 year was higher by smoking consumption and lower by alcohol consumption. Moving to the current smoking consumption of elderly people, there was an 8.7 years gap between Q1 and Q5 [Q1: 12.6 (CI 11.6–12.9) vs. Q5: 21.3 (CI 20.2–34.1)] followed by current tobacco consumption among the elderly people [Q1: 9.3 (CI 8.6–10.1) vs. Q5: 12.0 (CI 10.5–12.9)]. Furthermore, the total number of years lost with more than three disease accumulation patterns was observed for all behavioural characteristics among the elderly population in the state.

**Table 8:** Total numbers of years spent due to Single –Multiple lung diseases at age 75 years, by MPCE Wealth quintile, Tobacco, Alcohol, and Smoking consumption

	Wealth quintile	One disease (95% CI)	Two diseases (95% CI)	3 + diseases (95% CI)	Total (95% CI)
Overall	Q1	1.3 (0.9–2.3)	2.4 (2.0–3.1)	7.2 (7.5–11.2)	10.9 (11.5–13.2)
	Q2	3.7 (2.6–4.2)	4.6 (3.2–5.2)	7.8 (6.2–8.5)	16.1 (18.3–20.1)
	Q3	1.4 (0.5–1.9)	2.2 (2.0–2.6)	9.6 (7.4–11.9)	13.2 (12.6–13.8)
	Q4	0.6 (0.2–1.3)	1.3 (1.1–2.3)	8.1 (6.3–9.8)	10.0 (9.5–10.8)
	Q5	3.9 (2.6–4.2)	7.8 (6.5–8.6)	13.6 (11.3–14.6)	25.3 (24.8–25.9)
Tobacco consumption	Q1	0.6 (0.2–0.8)	1.2 (0.9–1.9)	7.5 (6.9–8.6)	9.3 (8.6–10.1)
	Q2	1.7 (1.0–2.2)	2.8 (2.2–3.6)	8.5 (7.3–12.3)	13.0 (12.6–13.6)
	Q3	1.8 (1.5–3.1)	2.5 (2.0–3.1)	4.9 (5.2–7.6)	9.2 (7.8–11.9)
	Q4	1.3 (0.9–1.9)	3.1 (2.5–3.9)	7.3 (6.6–8.5)	11.7 (13.2–14.9)
	Q5	1.5 (0.8–2.0)	2.7(2.0–3.0)	7.8 (6.9–8.6)	12.0 (10.5–12.9)
Alcohol consumption	Q1	0.9 (0.2–1.7)	2.3 (1.9–3.6)	3.4 (2.6–4.9)	6.6 (6.0–7.1)
	Q2	0.9 (0.2–2.1)	0.9 (0.2–1.6)	10.4 (9.8–11.9)	12.2 (11.8–12.6)
	Q3	1.0 (0.5–1.5)	2.1 (1.5–3.7)	8.5 (7.6–12.2)	11.6 (11.0–12.0)
	Q4	1.1 (0.8–1.9)	2.3 (1.5–4.3)	8.1 (7.1–9.5)	11.5 (9.5–14.6)
	Q5	2.6 (2.0–3.2)	3.5 (2.3–9.6)	5.5 (8.6–12.6)	11.6 (15.6–20.2)
Smoker consumption	Q1	1.4 (1.0–2.1)	2.1 (1.9–2.3)	9.1 (7.6–13.2)	12.6 (11.6–12.9)
	Q2	3.7 (2.6–4.1)	4.5 (3.2–6.9)	11.5 (10.2–16.3)	19.7 (18.6–21.6)
	Q3	1.5 (1.1–2.0)	2.1 (1.8–2.6)	8.2 (8.6–9.6)	11.8 (12.0–13.6)
	Q4	2.0 (1.1–2.5)	3.4 (2.6–4.9)	8.5 (7.2–9.3)	13.9 (11.0–14.9)
	Q5	4.1 (2.3–5.2)	8.3 (6.6–10.2)	8.9 (6.9–10.2)	21.3 (20.2–34.1)

## Discussion

The present study describes the prevalence of multiple lung diseases associated with wealth status and their behavioural factor among the West Bengal population aged people 45 years and above. The finding demonstrates that a strong positive gradient of age in the prevalence of lung diseases among older adults aged 45 years and above. The overall prevalence of lung diseases among this group was 8.21%. Among them 6.48% of older adults reported they have suffered from at least one lung disease and 1.81% from more than one lung diseases. Notably, the prevalence of lung diseases reported in this study was much lower than in previous studies conducted in Brazil [12], England [43], and Turkey [9]. A few studies conducted in India reported that the prevalence of lung diseases was higher among the aged 45 years than in ages above 70 years [44–47], but none of the studies were conducted on multiple lung diseases in the state. An umbrella country conducted the study and reported that high-income countries had a higher prevalence of lung diseases among the elder population [48–52]. The country variations point to lung disease heterogeneity within the state due to various demographics, socioeconomic, and sample characteristics in the study population. The result shows that the prevalence of lung diseases with one disease and more than one disease were higher among the male older adult than the female older adult. The higher prevalence of lung diseases among the elderly male can be attributed to the fact that male generally uses alcohol, smoke, and tobacco consumption. The previous study mentioned that the consumption of spirit was more highly associated with lung diseases among men than the other beverage type, while in women risk was associated with the consumption of beer than spirit or wine [49]. The prevalence of lung diseases was more marked in rural residents as compared to urban residents. The possible explanations for the higher prevalence of lung disease in rural residents include delayed medical treatment, low awareness, low education, and high out-of-pocket expenditure. Furthermore, public health services are often not client-friendly in rural areas because of variations in the timings, weak community participation in NGOs and CBOs, lack of doctors, and non-availability of medicines in government health facilities.

In the context of wealth status, lung diseases reflect a paradoxical pattern within the richest wealth quintile, higher level of education, and caste in the state. The previous work also reported that longer survival of the richest people documented a higher prevalence of lung diseases [10]. Another study also mentioned richest people have greater access to health care services than poorer people, thus there are more reports in lung diseases as compared to other economic background people [47]. Higher education plays the dominant role in decreasing the lung diseases prevalence among people in the state. The prior study mentioned that “an increased education linked to health care knowledge, affecting lifestyle behaviours reduced the risks of lung diseases” [53]. The prevalence of lung diseases was remarkably higher in Muslim communities and tribal populations. The reasons for the high risks of lung diseases among these communities have been attributed to various social and structural factors which included poverty, less coverage of health care facilities, low education, and lack of awareness on lung diseases, alcohol, smoking, and poor nutrition. Concerning working status, the non-working rich people had a higher prevalence of lung diseases than the poor non-working people. This might explain that the richest older adults are currently less engaged in physical activity because of their high socio-economic status, while in the poorest group, people are more active in the agricultural sector or any other primary sector which could be reflected in the lower prevalence rate [54]. The earlier study also mentioned the higher prevalence of lung diseases among the working elderly adult which could be attributed to occupation-based physical activity, more sitting time, and dietary level factors [55]. Furthermore, the richest economic people are more capable to purchase high-level alcohol and numerous types of cigarettes, thus prevalence of lung diseases, was higher among the richest elderly than the poorest elderly people.

Analysis of our main objective: the prevalence of lung diseases due to behavioural factors among elderly people in the state, the results showed alcohol, smoking, and tobacco consumption were significantly associated with lung diseases, although the prevalence of lung diseases was lower due to alcohol consumption than smoking and tobacco consumption. An earlier study found that “smoking is one of the most important predictive risk factors which is responsible for 70% of lung cancer-related mortality worldwide” [13]. While in China, smoking is not probably a risk factor for lung diseases among the Chinese older women, Chinese women are more exposed to second hand smoke, and high levels of household air pollution due to cooking with poor ventilation and stove, coal, charcoal, and other solid fuel [17]. As a result, they are more exposed to Asthma, Bronchitis, and other types of multiple lung diseases. However, the prevalence of multiple lung diseases in West Bengal was higher than in previous studies. Chennai reported a higher prevalence of lung diseases attributed to smoking, particularly among males. The correlation was significantly stronger among older adults aged 60 years and above compared to younger adults aged 45 years [30]. In Pune, a high prevalence of oral cancer was significantly associated with smoking bidi than cigarettes. Another interesting finding is the effect of wealth status on substance use of behavioural characteristics. Our results showed the prevalence of lung diseases was higher among the richest quintile due to high levels of alcohol consumption, while smoking and tobacco consumption highly affected lung diseases among the poorest quintile. This gradient might be reflected due to the wide variation of socio-economic quintiles. “Bidi smoking was observed to be more prevalent than a cigarette; this could be attributed to the cheap cost thereby implicating its affordability by the low socioeconomic status” [56].

The magnitude of individual socio-economic disparities in lung disease incidence and old age survival differs by cancer-related risk factors and behaviours such as consumption of alcohol, smoking, and tobacco. A strong income gradient in lung disease prevalence reflects a higher incidence of diseases among the richest household with long-time survival with morbidity [1]. The diseases-related treatment-seeking behaviour, lung disease diagnosis, and scanning facilities are better equipped and agglomerated in the richest household which could explain the long-time suffering from multiple diseases among the richest household. The prevalence of catastrophic expenditure is more concentrated among the poorest households leading to their dependence on treatment in public health care, while in the richest households, they rely more on private hospitals for inpatient care. A growing number of studies mentioned that lung cancer mortality rates were higher among the lower socio-economic group of people than higher socio-economic groups of people [57–61]. The largest inequalities of lung diseases prevalence were found between the poorest to richest wealth quintiles due to smoking and tobacco consumption. Franceschini reported, “smoking is a strong risk factor for lung cancer because it’s correlated with alcohol consumption” [12]. While another study mentioned the lower economic background of people suffering from lung cancer, and the survival time and survival rates are lower, which are not depending on individual or different area levels [44, 62].

### Limitations and strengths of the study

The study was based on self-reported disease information by the population which could be recall bias. As LASI data does not carry out the laboratory experiment on lung diseases measure, therefore it cannot be directly compared with clinical data. Secondly, the data does not provide information on the geographical climatic variation, and air pollution which has a strong association with lung diseases. Lastly, the LASI data did not cover the physical barrier such as transport facility, distance from the health centre, and quality of cigarettes and alcohol which may have resulted in variations between diseases.

Despite the limitations, the study provides reliable information on research designs and standardized data collection processes and awareness of lung diseases among the older population. Moreover, this is the first epidemiological study to investigate the multiple lung diseases prevalence and the number of years of life lost with disability in the state.

### Policy implication of the study

The findings of this study have significant policy implications for improving healthcare in West Bengal. Addressing the high prevalence of multiple lung diseases among the population requires targeted interventions, such as expanding healthcare access through subsidized services and financial assistance for economically disadvantaged individuals. Public health campaigns should prioritize behavioural change by raising awareness of the harmful effects of smoking and other risk factors contributing to lung diseases. Strengthening rural healthcare infrastructure with trained personnel and diagnostic facilities is essential for the timely detection and management of respiratory conditions. Continuous research and data monitoring will support evidence-based policymaking, ensuring sustainable long-term strategies. Additionally, policies should focus on increasing awareness of lung disease risk factors, reducing tobacco consumption, and overcoming fundamental barriers to provide equitable and affordable healthcare services, particularly for the elderly population across the state.

## Conclusion

In conclusion, the result of the study suggested that concomitant experience of single and multiple lung diseases is significantly associated with the age, wealth status, smoking, and tobacco consumption. In the state, the richest and poorest, both economic groups of people are vulnerable to long time lung disease due to smoking and tobacco consumption. In the context of lung diseases and behavioural factors, only education plays a significant role in cognitive function and health literacy, which can affect more awareness, and health-seeking behaviour and reduce the risk of disease incidence among elderly people in the state. For this issue, it will need to actively control the cancer prevalence in the older population. In addition, Government should be focused on early diagnosis, improvement of cancer surveillance, treatment, and scanning programs for rural and urban areas in the state.

## Data Availability

This research work was performed based on secondary data which is freely available upon request at IIPS, India website (Source of data: https://www.iipsindia.ac.in/lasi).

## References

[1] Rajpal S, Kumar A, Joe W. Economic burden of cancer in India: evidence from cross-sectional nationally representative household survey. PloS One. 2018;2014:1–17. 10.1371/journal.pone.0193320. 10.1371/journal.pone.0193320 PMC5826535 29481563

[2] Jones R, Kirenga B, Buteme S, Williams S, Gemert FV. Original Article A novel lung health programme addressing awareness and behaviour-change aiming to prevent chronic lung diseases in rural Uganda. Afr J Respir Med. 2019;14(2):2–9.

[3] World Health Organization. Asthma fact sheet, 31 August 2017. www.who.int/news-room/fact-sheets/detail/asthma.

[4] Dela Cruz CS, Tanoue LT, Matthay RA. Lung cancer: epidemiology, etiology, and prevention. Clin Chest Med. 2011;32(4):605–44. 10.1016/j.ccm.2011.09.001. 22054876 10.1016/j.ccm.2011.09.001 PMC3864624

[5] Sheth H, Kumar P, Limaye S. Management of metastatic nonsmall cell lung cancer in elderly. Indian J Med Paediat Oncol. 2021. 10.1055/s-0041-1732784.

[6] Cancer today. Available from: http://gco.iarc.fr/today/home. [Last accessed on 2022 Mar 27].

[7] Venuta F, Diso D, Onorati I, Anile M, Mantovani S, Rendina EA. Lung cancer in elderly patients. J Thorac Dis. 2016;8(1):908–14. 10.21037/jtd.2016.05.20. 10.21037/jtd.2016.05.20 PMC5124601 27942414

[8] Owonikoko TK, Ragin CC, Belani CP, Oton AB, Gooding WE, Taioli E, Ramalingam SS. Lung cancer in elderly patients: an analysis of the surveillance, epidemiology, and end results database. J Clin Oncol. 2007;25(35):5570–7. 10.1200/JCO.2007.12.5435. 18065729 10.1200/JCO.2007.12.5435

[9] Sari M. Current trends in the incidence of non-small cell lung cancer in Turkey : lung cancer aging. Eur J Med Investig. 2020;4(2):169–72. 10.14744/ejmi.2020.

[10] Arokiasamy P, Jain K. Multi-morbidity, functional limitations, and self-rated health among older adults in India: cross-sectional analysis of LASI Pilot Survey, 2010. Sage Open. 2015. 10.1177/2158244015571640.

[11] Zhong YJ, Wen YF, Wong HM, Yin G, Lin R. Trends and patterns of disparities in burden of lung cancer in the united states. Front Oncol. 2019. 10.3389/fonc.2019.00404. 31214489 10.3389/fonc.2019.00404 PMC6555199

[12] Franceschini JP, Jamnik S, Santoro IL. Survival in a cohort of patients with lung cancer: the role of age and gender in prognosis. J Bras Pneumol. 2017;43:431–6. 10.1590/S1806-37562016000000298. 29340491 10.1590/S1806-37562016000000298 PMC5792042

[13] Cinar D. Cancer in the elderly. North Clin Istanb. 2015;2(1):73–80. 10.14744/nci.2015.72691. 28058345 10.14744/nci.2015.72691 PMC5175057

[14] DeSantis CE, Miller KD, Dale W, Mohile SG, Cohen HJ, Leach CR, Sauer AG, Jemal A, Siegel RL. Cancer statistics for adults aged 85 years and older. Cancer J Clin. 2019;69:452–67. 10.3322/caac.21577. 10.3322/caac.21577 PMC12103238 31390062

[15] Pham J, Conron M, Wright G, Mitchell P, Ball D, Philip J, Brand M, Zalcberg J, Stirling RG. Excess mortality and undertreatment in elderly lung cancer patients : Treatment nihilism in the modern era ? ERJ Open Res. 2021. 10.1183/23120541.00393-2020. 34046489 10.1183/23120541.00393-2020 PMC8141829

[16] Kolk M, Jebari K. Sex selection for daughters : demographic consequences of female - biased sex ratios. Popul Res Policy Rev. 2022. 10.1007/s11113-022-09710-w.

[17] Pilleron S, Sarfati D, Janssen-Heijnen M, Vignat J, Ferlay J. Pilleron—global cancer incidence in older adults 2012 and 2035 a population-based study. Int J Cancer. 2019;144:49. 29978474 10.1002/ijc.31664

[18] Gupta PC, Sinha DN. Tobacco research in India. Indian J Public Health. 2004;48(3):103–4. 15709594

[19] Goswami A, Reddaiah VP, Kapoor SK, Singh B, Dwivedi SN, Kumar G. Tobacco and alcohol use in rural elderly Indian population. Indian J Psychiat. 2005;47(4):192. 10.4103/0019-5545.43050 PMC2921132 20711304

[20] Islami F, Goding Sauer A, Miller KD, Siegel RL, Fedewa SA, Jacobs EJ, Jemal A. Proportion and number of cancer cases and deaths attributable to potentially modifiable risk factors in the United States. CA Cancer J Clin. 2018;68(1):31–54. 10.3322/caac.21440. 29160902 10.3322/caac.21440

[21] Reddy KS, Shah B, Varghese C, Ramadoss A. Responding to the threat of chronic diseases in India. Lancet. 2005;366(9498):1744–9. 10.1016/S0140-6736(05)67343-6. 16291069 10.1016/S0140-6736(05)67343-6

[22] http://www.who.int/substance_abuse/facts/tobacco/en/print/html

[23] World Health Organization-Cancer Country profiles. World Health Organization. 2014.

[24] World Health Organization-Cancer Country profiles. World Health Organization. 2020

[25] World Health Organization. Reducing risks, promoting healthy life. World health report 2002. Geneva: WHO; 2002. 10.1001/jama.288.16.1974 12387638

[26] Bandera EV, Freudenheim JL, Vena JE. Alcohol consumption and lung cancer: a review of the epidemiologic evidence. Cancer Epidemiol Biomark Prev. 2001;10(8):813–21. 11489747

[27] Korte JE, Brennan P, Henley SJ, Boffetta P. Dose-specific meta-analysis and sensitivity analysis of the relation between alcohol consumption and lung cancer risk. Am J Epidemiol. 2002;155(6):496–506. 10.1093/aje/155.6.496. 11882523 10.1093/aje/155.6.496

[28] Kurmi OP, Lam KBH, Ayres JG. Indoor air pollution and the lung in low-and medium-income countries. Eur Respir J. 2012;40:239. 10.1183/09031936.00190211. 22362845 10.1183/09031936.00190211

[29] National Cancer Registry Programme. Two‐year report of the population based cancer registries 1997–1998. Incidence and distribution of cancer. Indian Council of Medical Research. 2002.

[30] Krishnamurthy A, Vijayalakshmi R, Gadigi V, Ranganathan R, Sagar TG. The relevance of “Nonsmoking-associated lung cancer” in India: a single-centre experience. Indian J Cancer. 2012;49(1):82–8. 10.4103/0019-509X.98928. 22842173 10.4103/0019-509X.98928

[31] Ganesh B, Talole SD, Dikshit R. A case-control study on diet and colorectal cancer from Mumbai. India Cancer Epidemiol. 2009;33(3–4):189–93. 10.1016/j.canep.2009.07.009. 19717354 10.1016/j.canep.2009.07.009

[32] Sehgal S, Kaul S, Gupta BB, Dhar MK. Risk factors and survival analysis of the esophageal cancer in the population of Jammu India. Indian J Cancer. 2012;49(2):245–50. 10.4103/0019-509X.102921. 23107978 10.4103/0019-509X.102921

[33] Halder P, Chattopadhyay A, Rathor S, Saha S. Nested multilevel modelling study of smoking and smokeless tobacco consumption among middle aged and elderly Indian adults: distribution, determinants and socioeconomic disparities. J Health Popul Nutr. 2024;43(1):182. 10.1186/s41043-024-00661-w. 39511693 10.1186/s41043-024-00661-w PMC11542357

[34] Upadhyay AK, Singh A, Kumar K, Singh A. Impact of indoor air pollution from the use of solid fuels on the incidence of life threatening respiratory illnesses in children in India. BMC Public Health. 2015;15:1–9. 10.1186/s12889-015-1631-7. 25884539 10.1186/s12889-015-1631-7 PMC4397688

[35] Halder P, Verma M, Pal S, Mishra AK, Deori TJ, Biswas R, Prabhakar MC. Association of anaemia with indoor air pollution among older Indian adult population: multilevel modelling analysis of nationally representative cross-sectional study. BMC Geriatrics. 2024;24(1):567. 10.1186/s12877-024-05171-2. 38951755 10.1186/s12877-024-05171-2 PMC11218345

[36] Kankaria A, Nongkynrih B, Gupta SK. Indoor air pollution in India: implications on health and its control. Indian J Commun Med. 2014;39(4):203–7. 10.4103/0970-0218.143019. 10.4103/0970-0218.143019 PMC4215499 25364142

[37] Pal J, Santra A. Disability among lung cancer patients and its predictors: a cross-sectional study in a tertiary care centre at Kolkata, India. Int J Res Med Sci. 2019. 10.18203/2320-6012.ijrms20192917.

[38] Longitudinal Ageing Study in India (LASI) Wave 1. International Institute for Population Sciences (IIPS), Mumbai, India, NPHCE, MoHFW, Harvard T. H. Chan School of Public Health (HSPH), and The university of Southern California (USC), 2020.

[39] Araujo MEA, Silva MT, Galvao TF, Nunes BP, Pereira MG. Prevalence and patterns of multimorbidity in Amazon Region of Brazil and associated determinants: a cross-sectional study. BMJ Open. 2018;8(11):e023398. 10.1136/bmjopen-2018-023398. 30391918 10.1136/bmjopen-2018-023398 PMC6231594

[40] Chang AY, Gómez-Olivé FX, Payne C, Rohr JK, Manne-Goehler J, Wade AN, Salomon JA. Chronic multimorbidity among older adults in rural South Africa. BMJ Global Health. 2019;4(4):e001386. 10.1136/bmjgh-2018-001386. 31423345 10.1136/bmjgh-2018-001386 PMC6688670

[41] Forrest LF, Sowden S, Rubin G, White M, Adams J. Socio-economic inequalities in stage at diagnosis, and in time intervals on the lung cancer pathway from first symptom to treatment: systematic review and meta-analysis. Thorax. 2017;72(5):430–6. 10.1136/thoraxjnl-2016-209013. 27682330 10.1136/thoraxjnl-2016-209013 PMC5390856

[42] Skyrud KD, Bray F, Eriksen MT, Nilssen Y, Møller B. Regional variations in cancer survival: impact of tumour stage, socioeconomic status, comorbidity and type of treatment in Norway. Int J Cancer. 2016;138(9):2190–200. 10.1002/ijc.29967. 26679150 10.1002/ijc.29967

[43] Syriopoulou E, Bower H, Andersson TM, Lambert PC, Rutherford MJ. Estimating the impact of a cancer diagnosis on life expectancy by socio-economic group for a range of cancer types in England. Br J Cancer. 2017;117(9):1419–26. 10.1038/bjc.2017.300. 28898233 10.1038/bjc.2017.300 PMC5672926

[44] Mallath MK, Taylor DG, Badwe RA, Rath GK, Shanta V, Pramesh CS, Sullivan R. The growing burden of cancer in India: epidemiology and social context. Lancet Oncol. 2014;15(6):e205–12. 10.1016/S1470-2045(14)70115-9. 24731885 10.1016/S1470-2045(14)70115-9

[45] Pal J, Santra A. Disability among lung cancer patients and its predictors: a cross-sectional study in a tertiary care centre at Kolkata, India. Int J Res Med Sci. 2019;7:2775. 10.18203/2320-6012.ijrms20192917.

[46] Komolafe A, Bhardwaj T, Kaushik A. Risk factors associated with lifestyle cancers in India: a systematic review of existing evidences from varied geographical locations. Clin Case Rep Int. 2022;6:1378.

[47] Mathew A, George PS, Ramadas K, Mathew BS, Kumar A, Roshni S, Booth CM. Sociodemographic factors and stage of cancer at diagnosis: a population-based study in South India. J Global Oncol. 2019;5:1–10. 10.1200/JGO.18.00160. 10.1200/JGO.18.00160 PMC6690651 31322993

[48] Śliwczyński A, Kalinka E, Sierocka A, Iltchev P, Kowalski D, Marczak M. Population morbidity in elderly lung cancer patients from Poland with specific trends in elderly women. Menopause Rev Przegląd Menopauzalny. 2019;18(3):161–5. 10.5114/pm.2019.90811. 10.5114/pm.2019.90811 PMC6970423 31975983

[49] Freudenheim JL, Ritz J, Smith-Warner SA, Albanes D, Bandera EV, Van Den Brandt PA, Hunter DJ. Alcohol consumption and risk of lung cancer: a pooled analysis of cohort studies. Am J Clin Nutrition. 2005;82(3):657–67. 10.1093/ajcn.82.3.657. 16155281 10.1093/ajcn.82.3.657

[50] Vaccarella S, De Vries E, Sierra MS, Conway DI, Mackenbach JP. Social inequalities in cancer within countries. Reducing social inequalities in cancer: evidence and priorities for research, 2019.

[51] Hovanec J, Siemiatycki J, Conway DI, Olsson A, Stücker I, Guida F, Behrens T. Lung cancer and socioeconomic status in a pooled analysis of case-control studies. PloS one. 2018;13(2):e0192999. 10.1371/journal.pone.0192999. 29462211 10.1371/journal.pone.0192999 PMC5819792

[52] Chauhan S, Patel R, Kumar S. Prevalence, factors and inequalities in chronic disease multimorbidity among older adults in India: analysis of cross-sectional data from the nationally representative longitudinal aging study in India (LASI). BMJ Open. 2022;12(3):e053953. 10.1136/bmjopen-2021-053953. 35351706 10.1136/bmjopen-2021-053953 PMC8961109

[53] Marengoni A, Angleman S, Melis R, Mangialasche F, Karp A, Garmen A, Fratiglioni L. Aging with multimorbidity: a systematic review of the literature. Ageing Res Revews. 2011;10:430–9. 10.1016/j.arr.2011.03.003. 10.1016/j.arr.2011.03.003 21402176

[54] Chauhan S, Kumar S, Nath NJ, Dosaya D, Patel R. Gender differential in chronic diseases among older adults in India: Does living arrangement has a role to play? Aging Health Res. 2022;2(4):100106. 10.1016/j.ahr.2022.100106.

[55] Seo S. Multimorbidity development in working people. Int J Environ Res Public Health. 2019;16(23):4749. 10.3390/ijerph16234749. 31783589 10.3390/ijerph16234749 PMC6926901

[56] Madani AH, Dikshit M, Bhaduri D. Risk for oral cancer associated to smoking, smokeless and oral dip products. Indian J Public Health. 2012;56(1):57–60. 10.4103/0019-557X.96977. 22684175 10.4103/0019-557X.96977

[57] Dabbikeh A, Peng Y, Mackillop WJ, Booth CM, Zhang-Salomons J. Temporal trends in the association between socioeconomic status and cancer survival in Ontario: a population-based retrospective study. Can Med Assoc Open Access J. 2017;5(3):E682–9. 10.9778/cmajo.20170025. 10.9778/cmajo.20170025 PMC5621958 28877916

[58] Denton EJ, Hart D, Russell PA, Wright G, Conron M. Lung cancer and socio-economic status: inextricably linked to place of residence. Intern Med J. 2017;47(5):563–9. 10.1111/imj.13376. 28105777 10.1111/imj.13376

[59] Ellis L, Coleman MP, Rachet B. The impact of life tables adjusted for smoking on the socio-economic difference in net survival for laryngeal and lung cancer. Br J Cancer. 2014;111(1):195–202. 10.1038/bjc.2014.217. 24853177 10.1038/bjc.2014.217 PMC4090723

[60] Wong M, Lao XQ, Ho KF, Goggins WB, Tse SL. Incidence and mortality of lung cancer: global trends and association with socioeconomic status. Sci Rep. 2017;7(1):1–9. 10.1038/s41598-017-14513-7. 29085026 10.1038/s41598-017-14513-7 PMC5662733

[61] Hiscock R, Bauld L, Amos A, Fidler JA, Munafò M. Socioeconomic status and smoking: a review. Ann N Y Acad Sci. 2012;1248(1):107–23. 10.1111/j.1749-6632.2011.06202.x. 22092035 10.1111/j.1749-6632.2011.06202.x

[62] Finke I, Behrens G, Weisser L, Brenner H, Jansen L. Socioeconomic differences and lung cancer survival—systematic review and meta-analysis. Front Oncol. 2018;8:536. 10.3389/fonc.2018.00536. 30542641 10.3389/fonc.2018.00536 PMC6277796

